# Effect of Maternal Body Mass Index on Hormones in Breast Milk: A Systematic Review

**DOI:** 10.1371/journal.pone.0115043

**Published:** 2014-12-23

**Authors:** Nicholas J. Andreas, Matthew J. Hyde, Chris Gale, James R. C. Parkinson, Suzan Jeffries, Elaine Holmes, Neena Modi

**Affiliations:** 1 Section of Neonatal Medicine, Department of Medicine, Chelsea & Westminster Hospital, Imperial College London, London, United Kingdom; 2 Section of Computational and Systems Medicine, Faculty of Medicine, Imperial College London, Sir Alexander Fleming Building, South Kensington, London, United Kingdom; German Diabetes Center, Leibniz Center for Diabetes Research at Heinrich Heine University Duesseldorf, Germany

## Abstract

**Background:**

Maternal Body Mass Index (BMI) is positively associated with infant obesity risk. Breast milk contains a number of hormones that may influence infant metabolism during the neonatal period; these may have additional downstream effects on infant appetite regulatory pathways, thereby influencing propensity towards obesity in later life.

**Objective:**

To conduct a systematic review of studies examining the association between maternal BMI and the concentration of appetite-regulating hormones in breast milk.

**Method:**

Pubmed was searched for studies reporting the association between maternal BMI and leptin, adiponectin, insulin, ghrelin, resistin, obestatin, Peptide YY and Glucagon-Like Peptide 1 in breast milk.

**Results:**

Twenty six studies were identified and included in the systematic review. There was a high degree of variability between studies with regard to collection, preparation and analysis of breast milk samples. Eleven of fifteen studies reporting breast milk leptin found a positive association between maternal BMI and milk leptin concentration. Two of nine studies investigating adiponectin found an association between maternal BMI and breast milk adiponectin concentration; however significance was lost in one study following adjustment for time post-partum. No association was seen between maternal BMI and milk adiponectin in the other seven studies identified. Evidence for an association between other appetite regulating hormones and maternal BMI was either inconclusive, or lacking.

**Conclusions:**

A positive association between maternal BMI and breast milk leptin concentration is consistently found in most studies, despite variable methodology. Evidence for such an association with breast milk adiponectin concentration, however, is lacking with additional research needed for other hormones including insulin, ghrelin, resistin, obestatin, peptide YY and glucagon-like peptide-1. As most current studies have been conducted with small sample sizes, future studies should ensure adequate sample sizes and standardized methodology.

## Introduction

Early life nutrition is considered a key candidate modulator of feeding behaviour, food intake and energy balance throughout life [1]. Evidence from a large number of observational studies identifies the neonatal period as a critical time for the long-term programming of adult health [2]. Meta-analyses indicate that breastfeeding, as opposed to formula feeding, may protect against the development of obesity and metabolic syndrome associated conditions in later life [3]–[5], though other research suggests that the association between formula feeding and obesity is explained by confounding [6]. Beneficial effects have been hypothesised to derive in part from the action of bioactive components in breast milk such as hormones [7]. To date, a number of hormones involved in the regulation of energy homeostasis have been identified in breast milk; these include leptin, adiponectin, insulin, ghrelin, resistin, obestatin, peptide YY (PYY) and glucagon-like peptide 1 (GLP-1) [8]–[15]. In addition to having a direct influence on infant appetite and weight gain, these peptides may also promote gut maturation and the development of neuronal circuits in the central nervous system that control metabolism. In this respect, hormones in breast milk may have long-term metabolic repercussions for the offspring following early life exposure [16].

In adults, the circulating concentration of these hormones are linked to an individual’s body weight and BMI; leptin is secreted primarily by adipocytes in proportion to the total amount of body adipose tissue, and is therefore positively correlated with BMI [17], as is resistin [18]. The concentration of serum insulin also increases with overweight and obesity [19]. In contrast, adiponectin [20], ghrelin [21], obestatin [22] and PYY [23] correlate negatively with adiposity and BMI, whilst the relation of GLP-1 with BMI is unclear [24]. Factors controlling breast milk hormone concentrations are also unclear. Whilst leptin, ghrelin, insulin and adiponectin have been speculated to pass from serum into breast milk [25]–[28], the mammary gland is also capable of synthesising various hormones [29], [30], potentially contributing to the quantity of hormone detected; the source of the other hormones investigated remains to be established.

The objective of this systematic review is to examine the concentration of appetite regulating hormones in breast milk, and their association with maternal BMI. We hypothesise that the concentrations of these hormones in breast milk correlate with maternal BMI.

## Subjects and Methods

### Literature search

A search in PubMed (www.ncbi.nlm.nih.gov) for studies published before 04/09/2014 in English was carried out using the following search terms and MEDLINE Medical Subject Headings (MeSH) terms (milk, human [MeSH] OR breastmilk OR “breast milk”) AND (leptin OR adiponectin OR resistin OR insulin OR ghrelin OR adipokine OR adipokines [MeSH] OR insulin [MeSH] OR ghrelin [MeSH] OR obestatin OR Peptide YY [MeSH] OR Glucagon-Like Peptide 1 [MeSH]). The literature search was conducted by NJA, assisted by MJH. For consideration into the systematic review the study must have included a report of breast milk concentrations of any of the hormones; leptin, adiponectin, insulin, ghrelin, resistin, obestatin, PYY and GLP-1, and their relation to maternal BMI, including pre-, in- or post-pregnancy BMI.

### Data extraction

Relevant studies were identified by evaluating the abstract, or by obtaining a full copy of the article if the abstract was not available. Review articles and commentaries were excluded. Reference lists of included articles were reviewed by the authors to identify further relevant studies. Data extracted included date of publication, number of participants, sample type, sample preparation method, measurement technique, time of sample collection post-partum, mean, standard deviation and range of maternal BMI, hormone concentration and the correlation coefficient between maternal BMI and hormone concentration. Data were independently verified by NJA and MJH and checked by CG. A PRISMA Checklist [31] was also completed to assist in the reporting of this systematic review (S1 Table).

### Quality of studies

A non-validated but pragmatic score by which to compare and measure the quality of included studies was devised. This involved assessing whether the study disclosed the type of sample analysed, whether maternal BMI was measured at the time of sample collection, if time of sample collection was standardised and adjustment was made for confounding factors and whether the study used appropriate statistical analysis. Sample preparation was considered appropriate if there was a centrifugation or sonication step prior to analysis. We considered that some kind of sample preparation was required to get a true reading of the hormone concentration, especially leptin. Previous research suggests that either leptin is associated with milk fat globules [29]; therefore samples require sonication in order to liberate leptin, or milk lipids interfere with the assays, so centrifugation is required [32]. Whichever the case, sample preparation appears to be needed in order to acquire accurate and reproducible readings. Sample size of less than 50 participants was defined as small, 50–100 as medium, and above 100 as large. The score was derived by totalling the number of factors the investigators had included and adding half a point for whether the study was small, one point for a medium sized study, and 1.5 points for a large study.

### Analysis of statistical methods used

As the concentration of hormones present in breast milk is non-normally distributed, non-parametric statistical analysis, or analysis of log transformed data was considered appropriate. Statistical significance was defined as a p value of below 0.05.

## Results

### Identified studies

**Figure 1:**
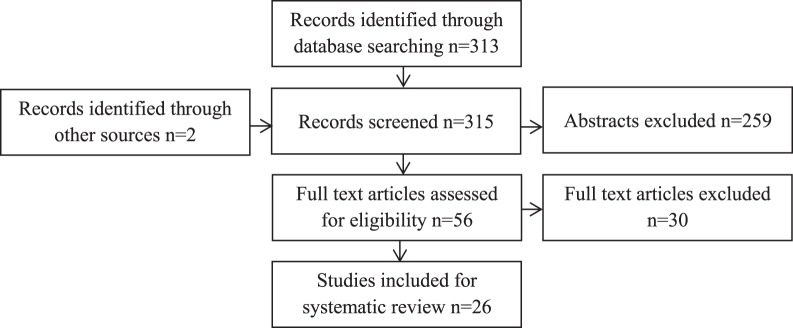
Flowchart of the search strategy used in the review. The relevant number of papers at each point is provided.

The literature search is outlined in **Fig. 1**. The search strategy identified 313 publications, with two further publications identified from reference lists. Abstracts were screened for suitability; 259 studies were unsuitable and excluded as breast milk hormone concentrations were not investigated. The full texts of 56 articles were reviewed; 30 studies were excluded after full-text review because they did not report the correlation between maternal BMI and breast milk hormone concentration, leaving 26 articles suitable for inclusion to the systematic review. The publications identified used a range of different techniques for quantification of hormone concentration, and reported concentrations over a wide range of time throughout lactation (see **Tables 1**, **2**, **3** **,** **4** and **5** for further details). **Table 6** gives an overview of the quality of the studies included. Studies investigating hormone concentrations in both colostrum and mature breast milk are evaluated. There was not a large difference seen in the concentration of hormone between these samples, however infants ingest significantly less colostrum than mature milk, whether there is a different influence of breast milk hormones in colostrum compared to mature milk is not known. Infants will ingest significantly greater quantities of hormone in mature milk; however, it may be that hormones in in colostrum are more active due to gut closure not having completed in colostrum fed infants [33].

**Table 1:** Studies examining the association between breast milk leptin concentrations and maternal BMI.

Author, Year	Maternal n number	Sample Type, Preparation and Analysis, Protease inhibitor used	Time of day, fasted/fed	Timing of Collection (day/week/month)	Maternal BMI (kg/m2)	Breast milk Leptin (ng/mL)			Statistical Analysis
Houseknecht, 1997	14	Complete breast expression. Skimmed and sonicated whole milk samples prepared. RIA. No protease inhibitor.	08:00am post-prandial	N/A	Not disclosed	Whole milk 10.1 ± 2.6*	Skimmed Milk 1.5 ± 0.9		Positive correlation seen with: Whole milk: r = 0.5, p<0.06 Skimmed Milk: r = 0.66, p<0.001
Ucar, 2000	18	Foremilk and hindmilk. Skimmed. RIA. No protease inhibitor.	Not disclosed	40 days post-partum	25.9±0.74, time not reported	3.36 ± 11.0			No correlation between breast milk leptin and maternal adiposity. Pearson’s correlation using log transformed values
Uysal, 2002	50	Foremilk. Analysis of Skimmed milk by RIA, No protease inhibitor.	08:00-11:00amNot disclosed	3 months post-partum	BMI at sample collection 25.9±4.4	Mothers of obese infants 0.27±0.2	Mothers of lean infants 0.37±0.4		Leptin concentrations were correlated with maternal BMI; r = 0.62, p<0.001 using Spearman correlation
Bielicki, 2004	33	Foremilk. Sonicated. RIA. No protease inhibitor.	Not disclosed.Fed	2-3 days post-partum 4-5 days post-partum 6 weeks post-partum	BMI taken at sample collection, 25.1±0.8, not reported, 23.6±0.85	2-3D = 1.15±0.12, 4-5D = 0.79±0.10, 6W not reported			Linear regression of log values. Correlation with maternal BMI: 2-3 days: r^2^ = 0.15, p = 0.02. No correlation found at 6 weeks
Dundar, 2005	AGA = 22 LGA = 14 SGA = 11	Foremilk. Sonicated, RIA, No protease inhibitor.	10:00-11:00am, not disclosed.	15 days post-partum 1 month post-partum 2 months post-partum 3 months post-partum	23.8 ± 0.77, time not reported	AGA 13.4±2.2 17.0±3.4 11.4±2.3 9.1±1.8	SGA 28.5±4.4 15.5±4.9 15.1±2.7 17.4±3.4	LGA 18.2±2.0 19.4±1.7 18.3±2.4 11.8±1.8	No relation between breast milk leptin and BMI. Pearson’s correlation
Bronsky, 2006	59	Hindmilk, skimmed. ELISA, EDTA tubes and protease inhibitor	7:00 am Not disclosed	48 hours post lactation	Pre-pregnancy 21.4±(0.4), time of delivery 26.8 ± (0.4)	0.5 ± 0.05			Correlation with: Pre-pregnancy BMI: r = 0.397, p = 0.003 BMI at delivery: r = 0.498, p<0.0001
Miralles, 2006	28	Hindmilk. Whole milk. ELISA No protease inhibitor.	Morning, Not disclosed	1±3 days post-partum 3 months post-partum 6 months post-partum 9 months ± 1 week	Pre-pregnancy, 21.6±0.5, range 16.3-27.3	1 month: 0.156 ± 0.039			Maternal BMI positively correlated with milk leptin concentration at each time point; r = 0.387, p<0.01. Stronger association found when concentrations were log transformed (r = 0.607). Pearson’s correlation
Weyermann, 2007	651	Foremilk. Analysis of skimmed milk samples by ELISA No protease inhibitor.	Not disclosed	33-71 days post-partum	Pre-pregnancy 23.6 ± 4.0 (16.7–45.7)	Median (Range): 0.175 (0-4.12), BMI <20: 0.122±0.129, BMI 20-24.9: 0.234±0.264, BMI 25-29.9: 0.388±0.335, BMI> 30: 0.807±0.885			Leptin concentrations were strongly associated with pre-pregnancy BMI
Bronsky, 2011	72	Hindmilk, skimmed. ELISA. EDTA tubes and protease inhibitor.	Not disclosed	0 day post lactation, 1 month, 3 months, 6 months, 12 months	Pre- pregnancy 21.9±0.4	0D = 0.3 ± 0.04, 1M = 0.2 ± 0.03, 3M = 0.1 ± 0.01, 6M = 0.1 ± 0.02, 12M = 0.2 ± 0.04			No correlation throughout the lactation period between pre-pregnancy BMI and leptin using Spearman’s correlation
Eilers, 2011	Term = 40 Preterm = 37	Type of milk not specified. Skimmed milk. RIA. No protease inhibitor.	16:00- 20:00 ‘No big meals’	Day 3 lactation, Day 28 lactation	Pre-pregnancy 23±3.5	Preterm, 3D = 0.7±0.79, 28D = 0.5±0.4	Term, 3D = 0.65±0.67, 28D = 0.5±0.4		Spearman’s correlation with pre-pregnancy BMI: Term: Day 3: r = 0.28, p<0.01; Day 28: 0.45, p<0.0001. Preterm: Day 3: r = 0.40, p = 0.016; Day 28: r = 0.56, p<0.01. Mothers with BMI>25 had higher leptin concentrations compared to those with a BMI <25, p<0.01
Schuster 2011	23	Sample sonicated and then skimmed. RIA. No protease inhibitor.	Not disclosed	1 week post-partum, 6 months post-partum	Pre-pregnancy 21.4±2.6, Median (IQR) 20.9 (19.3-22.6)	1W = 0.21±0.19, 6M = 0.18±0.15			Leptin concentration correlated with maternal BMI; r = 0.298, p<0.001. Pre-pregnancy BMI did not correlate with leptin concentration, Spearman’s correlation
Fields, 2012	19	Complete breast expression. Skimmed. Immunoassay. No protease inhibitor.	8:00-10:00am. Fasted 2h	40±4 days post-partum	Pre-pregnancy 26.6±6.6	0.092 ± 0.05			Positive Spearman’s correlation between pre-pregnancy BMI and leptin concentration; r = 0.78, p<0.0001
Savino, 2012	23	Sample sonicated and then skimmed. RIA. No protease inhibitor.	Not disclosed	87±40 days post-partum	Median (IQR) 27.4 (4.9), time not reported	Median (IQR): 2.34 (5.73)			No correlation between maternal anthropometric parameters and leptin using Spearman’s correlation
Schueler, 2013	13	Foremilk and hindmilk. Skimmed. RIA. No protease inhibitor used.	07:00-10:00am, fasted 10h	4-5 weeks post-partum	BMI at sample collection 25.9±4.23 Range 20.35-32.9	1.0 ± 0.7 Range 0.2-2.6			Leptin concentrations were positively correlated with maternal BMI, r = 0.82, p = 0.001. Pearson correlation
Brunner, 2014	6 weeks 152, 4 months 120	Complete breast expression. Sonicated then skimmed. RIA. No protease inhibitor used.	After overnight fast	6 weeks post-partum 4 months post-partum	Pre- and during pregnancy BMI. Values not reported	Median (IQR) 6W = 0.11 (0.19), 4M = 0.09 (0.18)			A strong positive correlation was observed between milk leptin and maternal pre-pregnancy BMI, 15wk and 32nd wk gestation BMI, r = 0.49, 052 and 0.57 respectively, p = <0.001 for all, at both time points, (6 week correlations shown). Spearman correlation

AGA: Appropriate for gestational age; EDTA: Ethylenediaminetetraacetic acid; ELISA: Enzyme-linked immunosorbent assay; IQR: Interquartile range; LGA: Large for gestational age; N/A: Not available; RIA: Radioimmunoassay; SGA: Small for gestational age.

*Data are given as means and standard deviations unless stated otherwise.

**Table 2:** Summary of findings of studies investigating leptin concentrations in breast milk.

	∼1 day	∼2 days	∼1 week	∼2 weeks	∼3 weeks	∼4 Weeks	∼6 weeks	∼2 months	∼3 months	∼6 months	∼9 months	∼12 months
**Ucar 2000**							**0**, NR					
**Uysal 2002**									**+**, r = 0.62, **p<0.001**			
**Bielicki 2004**		**+**, r^2^ = 0.15, **p = 0.02**	**+**, NR				0, NR					
**Brunner 2014**							**+**, r = 0.57 **p = <0.001**		**+**, r = ∼0.57 **p = <0.001**			
**Dundar 2005**				**0**, NR		**0**, NR		**0,** NR	**0,** NR			
**Bronsky 2006**		**+**, r = 0.498, **p<0.0001**										
**Miralles 2006**						**+**, r = 0.387, **p<0.01**			**+**, r = 0.387, **p<0.01**	**+**, r = 0.387, **p<0.01**	**+**, r = 0.387, **p<0.01**	
**Weyermann 2007**							**+, NR**					
**Eilers 2011**		**+,** r = 0.28, **p<0.01**				**+**, 0.45, **p<0.0001**						
**Bronsky 2011**	**0,** NR					**0**, NR			**0**, NR	**0**, NR		**0**, NR
**Schuster 2011**			+, r = 0.298, **p<0.001**	+, r = 0.298, **p<0.001**	+, r = 0.298, **p<0.001**	**+**, r = 0.298, **p<0.001**		+, r = 0.298, **p<0.001**	**+**, r = 0.298, **p<0.001**	**+**, r = 0.298, **p<0.001**		
**Fields 2012**						**+**, r = 0.78, **p<0.0001**						
**Savino 2012**									**0**, **NR**			
**Schueler 2013**						+, r = 0.82, **p = 0.001**						

Boxes with pluses indicate the study found a positive correlation between breast milk leptin concentration and maternal BMI at this time point. Boxes with zeros indicate the study found no correlation at this point. NR = Not reported, bold print indicates p value of <0.05.

**Table 3:** Studies examining the association between breast milk adiponectin concentrations and maternal BMI.

Author, Year	Maternal nNumber	Sample Type, Preparation andAnalysis Protease inhibitor used	Time of day,fasted/fed	Timing of Collection(day/week/month)	MaternalBMI (kg/m^2^)	Breast milkAdiponectin (ng/mL)	StatisticalAnalysis
Martin, 2006	22	Whole breast expression Skimmed. RIA. No protease inhibitor	Not disclosed	2–242 days post-partum	Post pregnancy 24.5 (19.5–34.2)	Median (Range): 17.7 (4.2–87.9)	Maternal post-pregnancy BMI was associated with natural log(milk adiponectin), β = 0.08±0.02, P<0.0001. This equates to an 8.33% increase in milk adiponectin concentration with each unit increase in maternal BMI
Weyermann, 2007	651	Foremilk. Analysis of skimmed milk samples by ELISA No protease inhibitor	Not disclosed	33–71 days post-partum	Pre-pregnancy 23.6 ± 4.0 (16.7–45.7)	Median (Range):10.9 (0.8–110), BMI<20: 12.8±9.7, BMI 20–24.9: 12.8±10.4, BMI 25–29.9: 11.8±9.1, BMI> 30: 14.8±12.1	Maternal pre-pregnancy BMI showed no clear association with adiponectin concentrations
Woo, 2009	45	Complete breast expression. Skimmed. RIA. No protease inhibitor	10:00 13:00, not disclosed	1 week post-partum, 2 weeks post-partum, 3 weeks post-partum, Then monthly	1 month post-partum 25.4 ± 3.5	25.6±8.4*	Milk adiponectin was log transformed and positively associated with maternal BMI (0.67±0.30 ng/ml increase in milk adiponectin per BMI unit, p = 0.02), effect not seen after adjusting for month or month^2^ (p = 0.11)
Dundar, 2010	25	Whole milk collected following an overnight fast. RIA No protease inhibitor.	08:00–10:00, not disclosed	1 day post-partum	Pre-pregnancy 23.1±0.8	29.5±6.4 Range: 1.26–77.1	No association between adiponectin concentrations and maternal anthropometric measurements using Spearman’s correlation
Bronsky, 2011	72	Hindmilk. Whole milk, ELISA, EDTA tubes and protease inhibitor	Not disclosed	0 day post-lactation, 1 month post-lactation, 3 months post-lactation, 6 months post-lactation, 12 months post-lactation	Pre- pregnancy 21.9±0.4	0D = 22.8±0.8, 1M = 22.0±0.6, 3M = 20.5±0.6, 6M = 21.4±0.8, 12M = 25.7±1.4	No correlation using Spearman’s correlation between maternal pre-pregnancy BMI and adiponectin
Luoto, 2011	181	Type of sample and preparation not reported. Colostrum samples analysed by dissociation enhanced lanthanide fluoro-immunoassay. No protease inhibitor	Not disclosed	0–3 days post-partum	Pre-pregnancy 23.8±3.7	Median (Range): 18.4 (2.9–317)	Pre-pregnancy BMI did not correlate with adiponectin concentration in colostrum (r = 0.023, p = 0.760). Log values
Luoto, 2011	30	Type of sample and preparation not reported. Colostrum samples analysed by dissociation enhanced lanthanide fluoro-immunoassay. No protease inhibitor	Not disclosed	1–3 days post-partum	Pre-pregnancy 23, range 18.40–28.37	Median (Range): 10.5 (3.1–98.9)	Regression analysis revealed no correlation between colostrum adiponectin concentration and pre-pregnancy maternal BMI
Ley, 2012	170	Foremilk. Skimmed. RIA No protease inhibitor	Not disclosed	2 days post-partum, 95 days post-partum	Pre-pregnancy 24.4 ±2.9	Median (IQR): 2D = 50.0 (21.9/104.6), 95D = 12.3 (9.9/17.2)	Pre-pregnancy BMI was not associated with adiponectin concentration in early or mature milk. General linear model, β ±SEE = 0.003 ±0.014, P = 0.81, in first week post-partum
Brunner, 2014	6 weeks 151, 4 months 120	Complete breast expression. Sonicated then skimmed. RIA. No protease inhibitor	After overnight fast	6 weeks post-partum, 4 months post-partum	Pre- and during pregnancy BMI. Values not reported	Median (IQR): 6W = 10.93 (8.34), 4M = 10.36 (9.40)	Using Spearman correlation, no correlation was observed between total milk adiponectin and maternal BMI, at either time point measured

EDTA: Ethylenediaminetetraacetic acid; ELISA: Enzyme-linked immunosorbent assay; IQR: Interquartile range; RIA: radioimmunoassay.

*Data are given as means and standard deviations unless stated otherwise.

**Table 4:** Studies examining the association between breast milk insulin concentrations and maternal outcomes.

Author, Year	Maternal n Number	Sample Type, Preparation and Analysis Protease inhibitor used	Time of day, fasted/fed	Timing of Collection (day/week/month)	Maternal BMI (kg/m^2^)	Breast milk Insulin (µIU/mL)		Statistical Analysis
Shehadeh, 2003	90	Milk type not reported. Skimmed. RIA. No protease inhibitor	Not disclosed	3 days and 10 days post-partum	23.7±5, time not reported	Day 3, 50.1±34.6* Median 41.0, Range 7-179	Day 10 41.1±28.5 Median 34.0, Range 12-183	Insulin concentrations were not significantly influenced by BMI on day 3 or day 10 post-partum. Spearman’s correlation
Ahuja, 2011	32	Complete breast expression, whole milk analysed by ELISA. No protease inhibitor	09:00-11:00 Not disclosed	6 weeks post-partum	Pre-pregnancy Non obese 21.8±2.34, Range 18.5-24.7, Obese 32.2±3.82, Range 26.4-40.2	Non-obese 4.5±7.6	Overweight/obese 30.1±56.3	There was a positive correlation between insulin concentration and maternal pre-pregnancy BMI (r = 0.57, p<0.001). Pearson’s correlation, non-logged values
Fields, 2012	19	Complete breast expression. Skimmed. Immunoassay. No protease inhibitor	8:00 -10:00am. Fasted 2h	40 ± 4 days post-partum	Pre-pregnancy 26.6±6.6	23.6±18.01		Insulin concentrations were not correlated with maternal BMI. Spearman’s correlation.
Ley, 2012	170	Foremilk. Skimmed. Electrochemilum-inescence immunoassay. No protease inhibitor	Not disclosed	2 days post-partum, 3 months post-partum	Pre-pregnancy 24.4 ±2.9	2D Median (IQR), 24.5 (9.2/57.9),	3M Median (IQR), 7.5 (4.9/12.5)	Higher pre-pregnancy BMI was associated with higher [insulin] in mature milk after adjustment for maternal age, ethnicity and time post-partum (β ± SEE: 0.053 ± 0.014; p = 0.0003) at 3 months, but not in the first week post-partum (β ± SEE: -0.002 ± 0.02; P = 0.91). General linear model

ELISA: Enzyme-linked immunosorbent assay; post-partum: post-partum; IQR: Interquartile range; RIA: radioimmunoassay.

*Data are given as means and standard deviations unless stated otherwise.

**Table 5:** Studies examining the association between breast milk ghrelin concentrations and maternal BMI.

Author, Year	Maternal n Number	Sample Type, Preparation and Analysis Protease inhibitor used	Time of day, fasted/fed	Timing of Collection (day/week/month)	Maternal BMI (kg/m^2^)	Breast milk Ghrelin (pg/mL)			Statistical Analysis
Aydin, 2006	Total study = 17 BMI analyses, number not stated “only a few participants examined”	Foremilk. Skimmed. RIA, no protease inhibitor.	Not disclosed	1 day post-partum, 7 days post-partum, 15 days post-partum	28.98±1.35, time not reported	1D = 73±18*, 7D = 84±18, 15D = 97±13			Spearman’s correlation between milk ghrelin concentrations and BMI r = 0.42, p = 0.19
Aydin, 2007	29	Foremilk. Skimmed. RIA and HPLC analysis, no protease inhibitor.	Morning, overnight fast	2 days post-partum, 15 days post-partum	Post-partum 29.6±1.9	RIA, Control, 2D = 64.1, 15D = 55.5, GDM, 2D = 26.1, 15D = 54.1, P-GDM, 2D = 28.5, 15D = 32.1,		HPLC, Active, 23±6, Non-active, 548±12, Total, 571±128	No correlation observed between ghrelin concentrations and post-partum maternal BMI
Aydin, 2010	20	Type of milk not reported. No sample preparation. ELISA. Protease inhibitor added	Not disclosed	Colostrum and mature milk	36.3±3.2, time not reported	ColostrumControl GDM Mature Milk Control GDM	Active 39.2±2 27.7±2 Active 48.2±5.1 27.7±2	Non-active 466.1±52 338.1±49 Non-active 505.1±52 359.1±51.2	Spearman’s correlation between fasting milk ghrelin concentrations and BMI was not significant r = 0.46, p = 0.6

ELISA: Enzyme-linked immunosorbent assay; GDM: Gestational Diabetes; HPLC: High Performance Liquid Chromatography; P-GDM: Pre-Gestational Diabetes; PP: post-partum; RIA: radioimmunoassay.

*Data are given as means and standard deviations unless stated otherwise.

**Table 6:** Table **6.** Table assessing the quality of study included into the systematic review.

	Stated what kind of milk	Measured BMI at time of sample collection	Standardised time post-partum of sample collection	Standardised time of sample collection	Adjusted for confounders	Appropriate Statistical analysis	Appropriate sample preparation	Small medium or large study	Score	Positive/negative/no correlation
**Leptin**										
Houseknecht, 1997	✓	✓	X	✓	X	X	✓	Small	4.5	Positive
Ucar, 2000	✓	✓	X	✓	X	✓	X	Small	4.5	No correlation
Uysal, 2002	✓	✓	X	✓	X	✓	✓	Medium	6	Positive
Bielicki, 2004	✓	✓	✓	✓	X	✓	✓	Small	6.5	Positive then none
Dundar, 2005	✓	Not stated	✓	✓	X	X	✓	Small	4.5	No correlation
Bronsky, 2006	✓	X	✓	✓	X	✓	✓	Medium	6	Positive
Miralles, 2006	✓	✓	✓	✓	X	✓	✓	Small	6.5	Positive
Weyermann, 2007	✓	X	X	Not stated	✓	✓	✓	Large	5	Positive
Bronsky, 2011	✓	X	✓	✓	X	✓	✓	Medium	6	No correlation
Eilers, 2011	X	X	✓	✓	X	✓	✓	Medium	5	Positive
Schuster 2011	X	✓	✓	X	X	✓	✓	Small	4.5	Positive
Fields, 2012	✓	X	✓	✓	X	✓	✓	Small	5.5	Positive
Savino, 2012	X	Not stated	X	✓	X	✓	✓	Small	3.5	No correlation
Schueler, 2013	✓	✓	✓	✓	X	X	✓	Small	5.5	Positive
Brunner, 2014	✓	X	✓	✓	X	✓	✓	Large	6.5	Positive
**Adiponectin**										
Martin, 2006	X	✓	X	✓	X	✓	✓	Small	4.5	Positive
Weyermann, 2007	✓	X	X	Not stated	✓	✓	✓	Large	5	No correlation
Woo, 2009	✓	✓	✓	✓	✓	✓	✓	Small	7.5	Positive, none after adjustment
Dundar, 2010	X	X	✓	✓	X	✓	X	Small	3.5	No correlation
Bronsky, 2011	✓	X	✓	✓	X	✓	✓	Medium	6	No correlation
Luoto, 2011	X	✓	✓	Not stated	X	✓	✓	Large	5	No correlation
Luoto, 2011	X	X	✓	Not stated	X	✓	✓	Small	3.5	No correlation
Ley, 2012	✓	X	✓	Not stated	X	✓	✓	Large	5	No correlation
Brunner, 2014	✓	X	✓	✓	X	✓	✓	Large	6.5	No correlation
**Insulin**										
Shehadeh, 2003	X	Not stated	✓	Not stated	X	✓	✓	Medium	4	No correlation
Ahuja, 2011	✓	X	✓	✓	X	✓	X	Small	4.5	Positive
Fields, 2012	✓	X	✓	✓	X	✓	✓	Small	5.5	No correlation
Ley, 2012	✓	X	✓	Not stated	X	✓	✓	Large	5	No correlation then Positive
**Ghrelin**										
Aydin, 2006	✓	Not stated	✓	✓	X	✓	✓	Small	5.5	Not significant
Aydin, 2007	✓	X	✓	✓	X	Not stated	✓	Small	4.5	No correlation
Aydin, 2010	X	Not stated	Not stated	Not stated	X	Not stated	✓	Small	1.5	Not significant

#### Leptin

Fifteen studies included a report of the correlation between breast milk leptin concentration and maternal BMI [8], [12], [14], [34]–[45] (**Table 1**). In ten studies, a positive correlation was reported at all time points measured [8], [14], [35], [38], [39], [41]–[45]. In an additional study a positive correlation at 2–3 days post-partum was found, but no correlation at six weeks post-partum [34]. In four studies no correlation was found between breast milk leptin and maternal BMI at any time point [12], [36], [37], [40]. In one study no correlation was identified between maternal BMI and colostrum leptin concentration [36]. In four studies a positive correlation was identified in the first two weeks of lactation between maternal BMI and breast milk leptin concentration [34], [35], [38], [44], whilst in one study there was no correlation at 15 days post-partum [37]. In five studies no correlation was found at 1–3 months post-partum [12], [34], [36], [37], [40], whilst in six studies a positive correlation was reported over the same period [14], [38], [39], [41]–[43]. In two studies a positive correlation was found up to six months post-partum [44], [45], whilst in a further study leptin concentrations were positively correlated with maternal BMI throughout the lactation period, up to nine months post-partum [39]. In one study no correlation was found at 12 months of lactation [36]. One study did not specify at which point samples were collected post-partum, but identified a positive correlation [8]. A negative correlation was not reported in any study (**Table 2**).

#### Adiponectin

Nine studies include a report of the concentration of adiponectin in breast milk and its association to maternal BMI [9], [36], [42], [45]–[50] (**Table 3**). Two studies report a positive correlation between breast milk adiponectin and maternal BMI [9], [50]. Woo *et al* [50] found a positive correlation between maternal BMI and breast milk adiponectin concentration. Samples were collected longitudinally, and due to a decrease in concentrations of adiponectin in samples collected later on in lactation, adjustment was made for post-partum month and month of the year, after which no correlation was observed. Martin *et al* [9] also reported a positive association between maternal BMI and breast milk adiponectin concentration. In seven studies, no correlation between maternal anthropometric measurements and milk adiponectin was found [36], [42], [45]–[49]. Many of the studies that found no correlation analysed colostrum rather than mature breast milk. Of the studies in which no correlation was found, six involved the analysis of colostrum [35], [36], [46]–[49]; in three studies samples were collected at 1–4 months post-partum [42], [45], [47], and in one study samples were collected up to 12 months post-partum [36].

#### Insulin

Four studies were identified in which the concentration of insulin in breast milk was measured, and the association with maternal BMI reported (**Table 4**). In two studies a positive correlation was reported; in two further studies no correlation was reported. Shehadeh *et al* [13] found no correlation on day three or day ten post-partum. Fields *et al* [43] found no correlation at one month post-partum. Ahuja *et al* [51] reported a positive correlation at six weeks post-partum whilst Ley *et al* reported a positive correlation between maternal BMI and breast milk insulin at three months post-partum, but not during the first week post-partum [47].

#### Ghrelin

Aydin and colleagues conducted all three studies identified in which the association between maternal BMI and breast milk ghrelin concentration was assessed [10], [52], [53]. A correlation was not reported in any of these studies (**Table 5**). In two studies, samples were collected at similar time points during the first two weeks post-partum [10], [53]; whilst the third neglected to state the time point post-partum in which samples were collected [52].

#### Resistin

A single paper by Savino *et al* in 2012 examined the association between maternal BMI and breast milk concentration of resistin. The median time of collection was 80 days post-partum. Median breast milk resistin concentrations reported were 0.18 ng/mL, with an interquartile range of 0.44. The author reported there was no correlation between breast milk resistin concentration and maternal anthropometric measurements [12].

#### Obestatin

No studies were identified in which the association between maternal BMI and breast milk obestatin concentration was examined.

#### Peptide YY

The association between breast milk concentration of peptide YY and maternal BMI was evaluated in a single paper by Schueler *et al* [14], the researchers also investigated breast milk leptin concentrations, and the study design is outlined in Table 1. Protease inhibitors were added to the aliquot used for peptide YY analysis. The researchers analysed peptide YY in both fore and hind milk, the average concentration in fore milk was 39.5±8.4 pg/mL (mean and standard deviation), whilst the hind milk concentration was 38.9±9.1 pg/mL at 4–5 weeks post-partum, as analysed by RIA. The authors reported that there was no correlation between maternal BMI and milk peptide YY.

#### Glucagon-like peptide 1

Concentrations of GLP-1 in breast milk were again investigated by Schueler *et al* [14], in the only study identified to do so. The study design is outlined in Table 1, protease inhibitor was used to preserve samples. In fore milk, the concentration of GLP-1 was 12.7±3.1 pM, whilst in hind milk it was 15.4±3.1 pM (mean ± SD). Milk GLP-1 was analysed using RIA for total GLP-1. The authors reported no correlation between maternal BMI and milk GLP-1.

## Discussion

This systematic review, which includes 26 papers and samples from over 1000 participants, indicates that there is an association between the concentration of leptin in breast milk and maternal BMI. Evidence for an association between maternal BMI and other hormones investigated is either contradictory or inconclusive. Inconsistencies in the data reported originate from the quality, design and size of studies. In addition to heterogeneous study design, many studies identified had small sample sizes, and hence limited power to detect differences in the concentration of hormones between mothers with differing BMI.

As BMI is not a direct measure of adiposity, the strength of the correlations between these hormones and BMI may not reflect the true value of this association. The increasing availability of more advanced techniques for evaluating body composition may facilitate such studies in the future. For example, to our knowledge, to date only one study has used dual X-ray absorptiometry to assess maternal adiposity [14]. A further limitation is non-standardised sampling; some studies involved analysis of either fore-milk or hind-milk, some a whole breast expression, and in some the sampling technique was not specified. This is potentially important as the concentration of hormones can change over an expression from a single breast, during a single feed [44] and with increasing duration of lactation [50]. Though this does not appear to be relevant for leptin [44], it is unknown whether this is the case for other hormones. In the majority of studies, samples were collected at a single time point, and those that collected data at multiple time points did not attempt to evaluate or adjust for time [42].

In regards to the methods of detection, predominantly immunoassay methods, RIA or ELISA were used. These techniques resulted in similar values, so it is unlikely that method of detection explains variability in the results obtained. More likely, the method of sample preparation contributes to the different results observed between different studies. In the majority of studies the supernatant of centrifuged breast milk was analysed, whilst in some, whole milk was used without prior treatment. There are conflicting recommendations about the most appropriate method of sample preparation. Some suggest that milk lipid interferes with the assays [32], whist others suggest it does not [8].

Many studies measured maternal BMI at different times during lactation or pregnancy. There does not appear to be any consistent variation in results obtained from studies measuring BMI at different time points, pre or post-partum. This suggests the most appropriate time to correlate concentrations of hormones in breast milk to maternal BMI is at the time of sample collection in order to reflect current maternal adiposity and hence maximise the likelihood of identifying an association. Furthermore, the time of sample collection could have a significant impact on results obtained. This is because concentrations of hormones change throughout the lactation period [50]. The majority of studies accounted for this by collecting samples at a specific time point, and those that did not adjusted for this effect [42].

**Table 2** displays the associations observed by different studies over the study period, all but one of these report consistent results over the study period. This could be due to either a consistent biological observation, or the methods of the studies being consistent.

Early nutrition may influence health outcomes in children and adults through metabolic programming; breastfeeding is associated with altered adiposity in infancy [54] and reduced overweight or obesity in later life compared to formula feeding [55], but evidence of a causal association is uncertain. In the PROBIT study, a cluster-randomized trial promoting breastfeeding, no difference was found in adiposity measures at 11.5 years in two groups fed differing amounts of breast milk [56]. Nonetheless, it is plausible that breast milk hormones might mediate offspring metabolism and risk of later obesity. This would require the presence of appetite regulating hormones in breast milk which retain their biological activity following ingestion, and that they affect infant metabolism. Supporting evidence comes from both human and animal models.

Previously, a positive correlation has been observed between maternal BMI, weight and maternal serum leptin concentration [57]. This appears to be reflected in breast milk, due to the positive correlation observed between breast milk leptin concentration and maternal BMI. Infant serum leptin and maternal BMI have also been shown to correlate, suggesting infants breast fed by mothers with a high BMI are exposed to significantly higher quantities of leptin, which is subsequently transferred to the infant bloodstream [58]. Infants born to obese mothers have been shown to be more likely to become obese themselves [59]. Potentially, the infant of the obese mother, who is ingesting an increased amount of leptin, becomes leptin resistant and subsequently has impaired appetite regulation, with an increased risk of obesity. Conversely increased leptin ingestion may enhance satiety, reducing intake and later obesity. However, confounding influences must be considered, such as the shared environment of mother and infant. Leptin present in milk may provide a link between maternal body composition and neonatal growth, development and energy balance. Further human studies have found correlations between the growth of neonates and the concentration of breast milk hormones [25], [39], [41].

Similarly to leptin, maternal serum adiponectin concentrations correlate with maternal weight and BMI [60], also breast milk adiponectin has been reported to be positively correlated with both maternal and infant serum adiponectin concentrations [50], [60], although it is present at significantly lower concentrations [27]. This suggests either the mammary epithelial cells are capable of synthesizing adiponectin, or are able to transfer adiponectin from the blood.

The quantity of adiponectin in breast milk may have biological implications for the infant; negative correlations between breast milk adiponectin concentration with infant and childhood adiposity have been found. Evidence demonstrates adiponectin in breast milk is associated with a lower weight of the infant in the first six months of life [61]. Furthermore, Luoto *et al* reported that colostrum adiponectin concentrations were significantly higher in mothers whose offspring were of normal weight at 10 years compared to mothers whose offspring were overweight at 10 years [49]. Further supporting evidence is the recent identification of adiponectin receptors in the human intestine [62]. The correlations observed between breast milk adiponectin concentrations and infant adiposity strengthens evidence suggesting there is an association between breastfeeding and a reduced risk of obesity in adulthood. This suggests that breast milk adiponectin is systemically absorbed in human infants, remains biologically active and is capable of programing infant metabolism. However, important confounding factors must be accounted for, as causality has not yet been demonstrated, this association may be a reflection of the increased adiponectin concentrations in normal weight mothers, who are more likely to have normal weight children.

Physiological doses of orally administered human leptin have been demonstrated to be absorbed into the bloodstream of nine day old rats [63]. Oral doses of leptin have been shown to have demonstrable biological effects, affecting food preference, body weight, i and reducing caloric intake and is capable of improving insulin sensitivity [64]–[67]. Likewise, evidence suggests exogenous insulin is able to be absorbed from the gut into the systemic circulation in rat models [68], retaining its biological activity and potentially mediating effects on infant body composition. In type 1 diabetic humans, all of the insulin present in the milk was artificial, also insulin in human milk is present at comparable concentrations to serum, suggesting there is an active transport mechanism [28]. Thus, current evidence supports the possibility of an association between enteral absorption of breast milk hormones with systemic effects, consistent with the hypothesis that breast milk hormones are an important factor in the programming of infant metabolism in the post-partum period [69].

The data presented here supports a correlation between increasing maternal BMI and increasing breast milk leptin. In the majority of studies a positive correlation between breast milk leptin and maternal BMI was found. In all but two of the studies less than 100 participants were recruited. However, a large study with 651 participants [42] demonstrated a strong association between maternal BMI and breast milk leptin concentration.

A correlation was not found between adiponectin concentrations in breast milk and maternal BMI. This is unexpected as in the serum of adults, adiponectin concentrations are known to be inversely proportional to adiposity [20]. Therefore, it would be logical that overweight and obese mothers would have less adiponectin present in their breast milk. In the study which did find an association between maternal BMI and the concentration of adiponectin in breast milk, a possible explanation is the association between adiponectin, prolactin and adiposity. Adiponectin is negatively correlated with prolactin [70], as prolactin secretion is reduced in obesity, if adiponectin is produced by the adipose tissue of the mammary gland, negative regulation by prolactin in more adipose women could increase the concentration of adiponectin produced in the mammary tissue, and secreted into breast milk. Although adiponectin in breast milk must be regulated by factors other than maternal BMI, such as duration of breastfeeding [50] and smoking status [42].

Evidence of a correlation between maternal BMI and breast milk insulin concentration was inconclusive. This is also unexpected; research investigating the breast milk of diabetic mothers found a direct correlation between serum and breast milk insulin concentrations [71], and it is well documented that the concentration of insulin in serum increases with increasing BMI, due to insulin resistance [72]. One potential explanation is that included studies are underpowered to detect this association.

With regard to ghrelin, conflicting results are also reported. Ghrelin can be either acylated or de-acylated. The acylated form has caprylic acid attached to a serine residue, and is commonly denoted as the active form, however, de-acyl ghrelin has also been shown to have appetite stimulating effects [73]. Acylated ghrelin is known to be particularly labile, and therefore it is especially important to either acidify samples at collection, or use a protease inhibitor if attempting to measure acylated ghrelin [74]. A positive correlation might be expected as the serum concentration of active ghrelin is increased in obese compared to normal weight patients [75], but this depends on the source of ghrelin found in breast milk. There have been conflicting reports in regard to this, with both maternal serum and mammary gland synthesis being suggested as the source of milk ghrelin [10], [30]. Correlations have been observed between maternal serum ghrelin concentration and breast milk ghrelin with infant serum concentrations, suggesting breast milk is a source of ghrelin for the infant [76].

Resistin concentrations increase with obesity, therefore concentrations of breast milk resistin might be expected to increase with maternal BMI. However, this inference is not supported by the study undertaken by Savino *et al* in which no correlation was found between maternal BMI and the concentration of resistin in breast milk [12]. However, the small number of women included means the study may be underpowered to examine this association.

Concentrations of both Peptide YY and Glucagon-like peptide 1 and their association with maternal BMI were evaluated in a single study. Neither of these hormones was found to have an association with maternal BMI. In regard to GLP-1, no clear association between BMI and serum concentrations of this hormone are yet to be established, therefore it is unsurprising an association was not identified in milk, where the association is likely to be less pronounced. In regard to PYY, where in serum, a negative association has been identified extrapolation to the breast milk suggests that concentrations would be decreased in obese mothers, unless there is another factor with a stronger influence on the concentration of this hormone in breast milk.

In summary, increasing maternal BMI is associated with an increase in the concentration of leptin in breast milk. No evidence of a correlation between maternal BMI and other hormones (adiponectin, insulin, ghrelin, resistin, obestatin PYY or GLP-1) was found, but published data are limited and interpretation problematic due to heterogeneity of study design, sample collection and preparation, and the small number of participants. Improved understanding of the potential of breast milk to influence offspring health requires that future studies address these important methodological issues. Future studies should ensure they are sufficiently sized, with *a priori* power calculations. Careful attention should be paid to define methods of breast milk collection, specifying whether fore and/or hind milk, or an entire expression was collected, and the use of protease inhibitor to prevent break down of peptide hormones is recommended [74]. Recording maternal BMI at time of sample collection is also recommended, to best reflect current maternal adiposity. To control for potential diurnal variations and changes over the course of lactation, sample collection should be carried out at a consistent time and day post-partum.

## Supporting Information

S1 Table PRISMA 2009 Checklist, completed to assist in the proper reporting of this systematic review.
(DOC)
